# The Importance of Differentiating Parsonage-Turner Syndrome From Cervical Radiculopathy: A Case Report

**DOI:** 10.7759/cureus.28723

**Published:** 2022-09-03

**Authors:** Ben Silverman, Tejas Shah, Gurtej Bajaj, Michael Hodde, Adrian Popescu

**Affiliations:** 1 Physical Medicine and Rehabilitation, University of Pennsylvania, Philadelphia, USA

**Keywords:** brachial neuritis, brachial plexopathy, shoulder weakness, cervical radiculopathy, parsonage-turner syndrome

## Abstract

Parsonage-Turner syndrome (PTS) is a rare disease process in which one develops acute-onset shoulder pain, followed by progressive weakness of the upper arm and shoulder girdle musculature. PTS is difficult to diagnose as it mimics similar presenting pathologies, most commonly, cervical radiculopathy (CR). Clinical presentation and diagnostic tests are particularly important to identify this rare syndrome, as the treatment for similar conditions may be more invasive. We present an interesting case of a 32-year-old female with severe unilateral shoulder pain, followed by weakness of her upper extremity musculature. The etiology of her symptoms cannot be concluded for certain; however, the aim of this case report is to increase awareness of this rare but potentially debilitating syndrome while also educating providers on the importance of differentiating PTS from the more commonly diagnosed CR.

## Introduction

Parsonage-Turner syndrome (PTS) was reported as a distinct diagnosis in 1948 when Parsonage and Turner described 136 cases of patients with sudden-onset shoulder pain, followed by flaccid paralysis of the shoulder girdle as the pain resolved [1]. The incidence is thought to be around two per 100,000 people, though typically under-reported given the difficulty in making the diagnosis [2]. Most cases occur between the ages of 20 and 60 years, with a male-to-female ratio of 2:1. Viral infections and vaccinations have been found to be precipitating factors in 25% and 15% of cases, respectively. The etiology is believed to be secondary to a viral illness that has direct effects on the brachial plexus or from an autoimmune response following a recent infection or immunization [3]. PTS is clinically diagnosed; however, certain diagnostic tests including electromyography (EMG) are used to confirm the clinical suspicion. This syndrome is typically self-limiting with patients having near-complete neurologic recovery at the end of three years [4]. Given the likelihood of self-resolution, it is crucial to avoid misdiagnoses, which can result in unnecessary procedures and potentially prolong patient suffering.

## Case presentation

**Figure 1:**
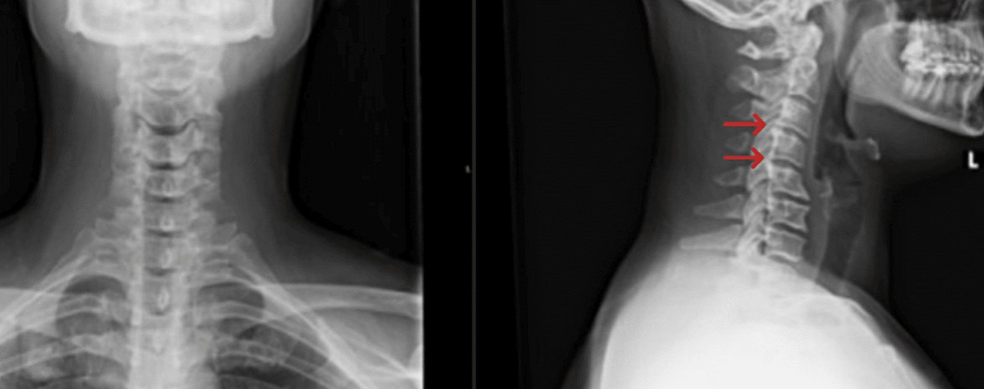
Cervical spine radiographs demonstrating straightening of the normal cervical lordotic curvature, multilevel degenerative changes with prominent anterior spurring, and notable foraminal narrowing at C3-C4 and C4-C5 (red arrows) L: left.

A 32-year-old female presented to an outpatient spine center with right upper extremity (RUE) weakness. Past medical history was notable for Tourette’s syndrome, attention-deficit/hyperactivity disorder, and anxiety. She had severe neck and shoulder pain that had begun one month prior, lasted one to two weeks, and ultimately resolved following a course of oral steroids. This painful phase was immediately followed by persistent right arm and shoulder weakness that worsened as the day progressed. There were no reported inciting or traumatic events other than a possible viral infection in the month leading up to her symptoms. Review of systems was notable for weakness, numbness, and tingling of the right shoulder and right thumb. She denied any recent fevers, chills, night sweats, falls, or episodes of incontinence. A physical exam showed a pleasant young female in no acute distress. There was no tenderness to palpation over the paraspinal musculature or spinous processes on musculoskeletal examination. The right shoulder had a full range of motion, and provocative tests specific for shoulder pathology were unremarkable. Neurological exam revealed normal reflexes, intact gait, and a negative Spurling’s and Tinel’s test. Utilizing the Medical Research Council (MRC) grading system, her RUE strength was noted to be a 4+/5 with shoulder abduction, 3/5 with elbow flexion, 4/5 with internal and external rotation, and 4/5 with supination. Cervical spine radiographs demonstrated straightening of the normal cervical lordotic curvature, multilevel degenerative changes with prominent anterior spurring, and notable foraminal narrowing at C3-C4 and C4-C5 (Figure 1). 

**Figure 2:**
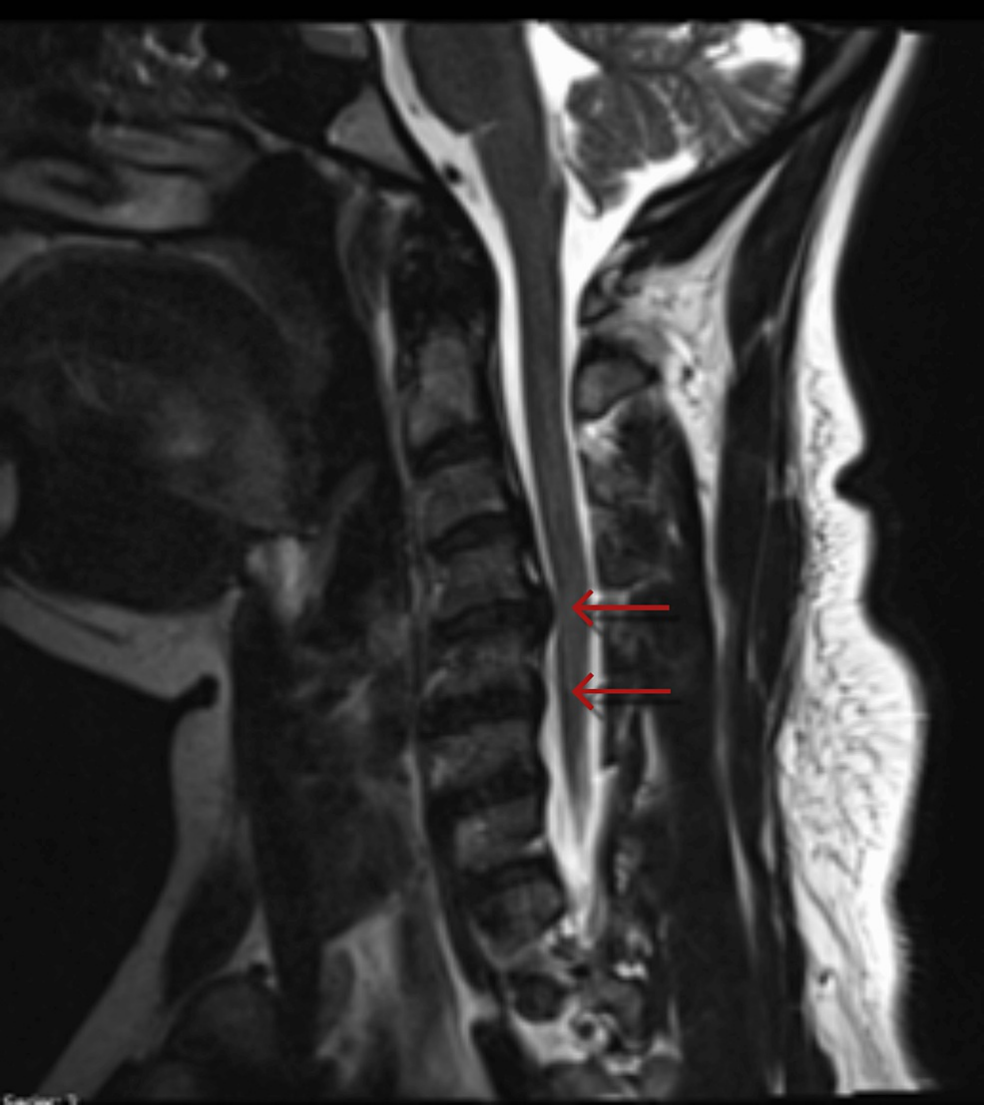
T2-weighted sagittal MRI of the cervical spine demonstrating moderate and mild disc bulges at C4-C5 and C5-C6 (red arrows), respectively

**Figure 3:**
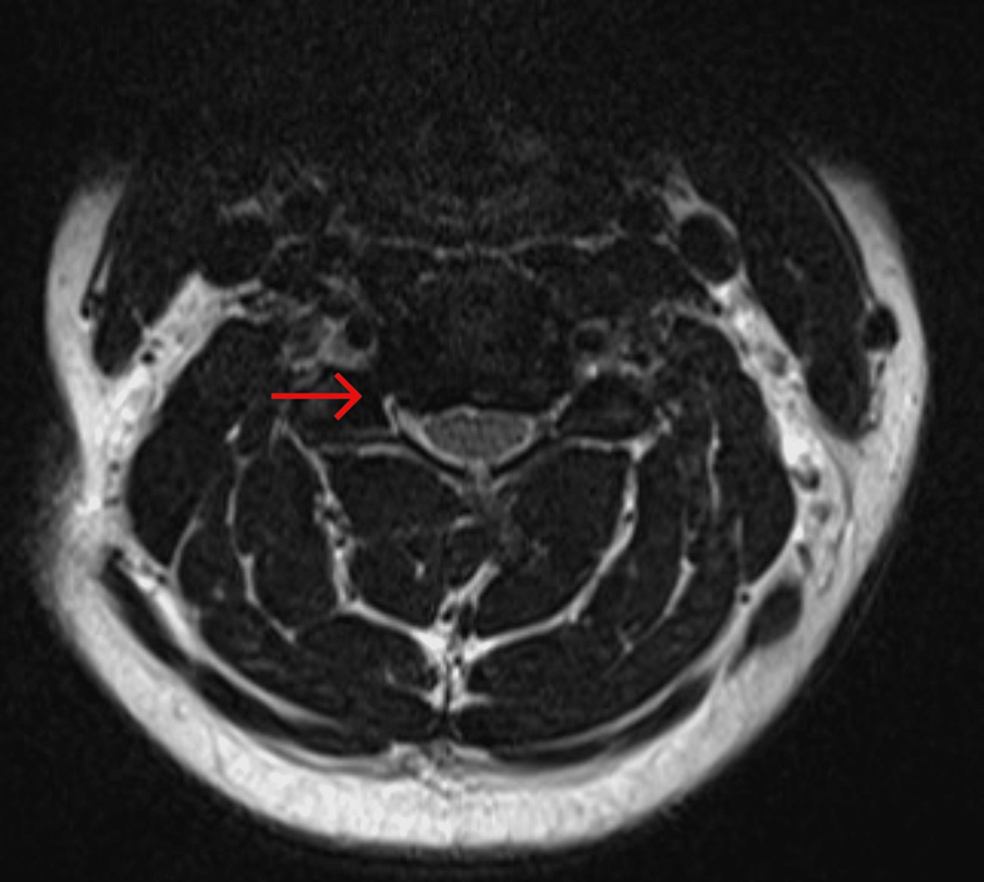
T2-weighted axial MRI of the cervical spine demonstrating severe right-sided foraminal stenosis at C4-C5 (red arrow)

T2-weighted magnetic resonance imaging (MRI) of the cervical spine demonstrated a moderate diffuse disc bulge, mild central canal stenosis, and moderate to severe bilateral neural foraminal narrowing at C4-C5 (Figures 2, 3).

The initial plan was to hold off on any spinal injections given that the patient's symptoms consisted only of a painless weakness. There was also the additional high risk of iatrogenic injury secondary to her Tourette’s syndrome. Electrodiagnostic studies (EDx) and a prescription for physical therapy were ordered. EMG results demonstrated severe chronic active right C5 radiculopathy. There was abnormal spontaneous activity seen in all C5-C6 innervated muscles tested, which included the deltoid, biceps brachii, and brachioradialis. Nerve conduction studies (NCS) were normal, which included the lateral antebrachial cutaneous nerve (LAC). After two months of physical therapy, she continued to have persistent RUE weakness and underwent repeat imaging which again demonstrated severe bilateral foraminal stenosis from C4-C6, worse at C4-C5. Cervical radiculopathy (CR) and PTS were the two leading diagnoses at this time; however, CR was ultimately deemed the official diagnosis given the EMG findings consistent with a C5 radiculopathy and MRI findings notable for severe right C4-C5 foraminal stenosis. After discussions with neurosurgery, the patient underwent a total disc replacement (TDR) at C4-C5 to try and relieve her symptoms. Follow-up visit one month post-TDR revealed unchanged RUE weakness. The physical exam at this visit revealed an MRC grade of 4-/5 throughout the RUE muscle groups, which was similar to her pre-surgical strength exam. She was instructed to continue physical therapy and follow-up in two months with neurosurgery.

## Discussion

PTS and CR are similar presenting pathologies; however, the latter is more commonly diagnosed. Both conditions are characterized by unilateral neck, shoulder, and upper extremity pain, often with associated sensory abnormalities and weakness. Furthermore, both conditions commonly occur without antecedent trauma. PTS tends to present as a more acute process compared to the more insidious onset of CR, although it is not uncommon to see an acute onset of CR [5]. The timeline in which the pain and weakness begin may serve as an important clinical distinction between these two conditions. The weakness in patients with PTS will usually start days to weeks after the acute painful phase has resolved, whereas the pain and weakness in radiculopathy will likely present simultaneously. The most common distinguishing features can be summarized in Table 1 [6].

**Table 1:** Common features of CR and PTS EMG: electromyography, MR: magnetic resonance, CR: cervical radiculopathy, PTS: Parsonage-Turner syndrome.

	Cervical Radiculopathy	Parsonage-Turner Syndrome
Onset	Typically more insidious than acute	Rapid and without antecedent trauma
Weakness timeline	Weakness may coincide with pain	Weakness will start days to weeks after the resolution of painful phase
Common presenting symptoms	Unilateral shoulder pain that radiates to the neck and often down the extremity	Unilateral shoulder girdle pain that radiates to the neck and upper extremity
Exacerbation	Exacerbated with neck movements; patients typically have a positive Spurling maneuver	Not exacerbated with neck movements; patients will have a negative Spurling maneuver
EMG findings	Denervation correlating to a specific root level	Denervation most commonly affecting the upper trunk of brachial plexus
MRI findings	Degenerative changes (bone spurs, disc changes) affecting a specific nerve root	Generally no specific localized finding; MR neurography may show constrictions of specific nerves

This case illustrates a patient who presented to an outpatient clinic with a clinical picture indicative of PTS; however, the MRI and EDx were consistent with CR. It can be difficult for a provider to conclude a diagnosis of CR or PTS when faced with mixed history and imaging results. In cases where the diagnosis may not be certain, examination of select muscle groups and specific diagnostic testing can be of utmost importance. A study by Scalf et al. concluded that MRI is sensitive for detecting signal abnormalities in the affected muscles of PTS, specifically intramuscular denervation changes including edema and atrophy [6]. This study also found that the supraspinatus and infraspinatus were the most involved muscles in imaging. A study by Stefanoff et al. found that more advanced imaging, specifically magnetic resonance neurography (MRN), has been shown to detect hourglass-like constrictions of nerves that are affected in PTS, an anomaly that is unique to patients with this syndrome [7]. This study found bilateral and multifocal nerve affection of the brachial plexus in all 17 studied patients who had undergone MRN, suggesting that this diagnostic test may be highly accurate in the diagnosis of PTS.

EDS can also be an important extension of the physical exam to further narrow in on the correct diagnosis. Muscles and nerves that are not commonly tested with EDS, specifically the supraspinatus and infraspinatus muscles, and the lateral antebrachial cutaneous nerve (LAC), can help differentiate the two conditions. The suprascapular, long thoracic, and axillary nerves have been documented in the literature to be the most affected peripheral nerves in PTS [8,9]. The LAC has been shown to be involved in around 32% of patients [10]. Paraspinal muscles are usually spared, as the roots of the brachial plexus are typically not involved. It should be noted that although this patient had a weakness with internal and external rotation on the physical examination, the supraspinatus and infraspinatus muscles were not tested on EMG due to operator inexperience.

The difference in treatment of PTS and CR ranges from conservative to surgical options. Conservative options for treating PTS include a combination of pain management and rehabilitation. Interestingly, a small study by the Korean Orthopedic Association proposed that steroids can improve recovery time and faster return to daily life when used specifically in the weakness phase [11]. A study by Tsairis et al. determined that patients typically have a good prognosis following a diagnosis of PTS [4]. This study found that 36% of patients had full functional recovery within one year, 75% by two years, and 89% by three years. Rarely will patients need surgical intervention for PTS; however, a study by Krishnan determined that neurolysis surgery can be considered for patients with PTS who have failed conservative treatment for 12 months or longer [12].

The treatment of CR is comparable to PTS in that many patients can improve with conservative measures, specifically physical therapy. Despite success with conservative management, many patients continue to be burdened by prolonged symptoms. Worsened or continued symptoms following conservative measures may prompt patients to seek more invasive management including an epidural steroid injection or even surgical intervention. The most common surgical intervention for CR is an anterior cervical diskectomy and fusion (ACDF). According to a study by Epstein, this procedure does have morbidity rates ranging from 13.2% to 19.3% [13]. This study also found that the most common complications following an ACDF included dysphagia, post-operative hematomas, exacerbation of myelopathy, recurrent laryngeal nerve palsy, and increased radicular symptoms. Readmission rates for ACDF ranged from 5.1% at 30 days to 7.7% at 90 days post-operatively. Therefore, it is all the more important to distinguish these two similar presenting pathologies and establish the correct treatment route to avoid unwanted complications. Although this patient was ultimately diagnosed with a C5 CR and underwent TDR, the lack of strength improvement at one-month follow-up continues to stress the notion that the diagnosis of PTS cannot be fully excluded. Continued follow-up will be needed to assess further recovery.

## Conclusions

This case report highlights the importance of obtaining a detailed history, performing a comprehensive strength examination, and ordering proper electrodiagnostic testing when faced with the similar presenting pathologies of PTS and CR. Being able to differentiate between these two conditions may limit unwarranted surgical procedures, surgical complications, and healthcare costs. PTS should always remain on the differential in patients who present with unilateral shoulder pain and weakness.
